# Invertebrate models of lamin diseases

**DOI:** 10.1080/19491034.2018.1454166

**Published:** 2018-04-16

**Authors:** Ryszard Rzepecki, Yosef Gruenbaum

**Affiliations:** a Laboratory of Nuclear Proteins, Faculty of Biotechnology, University of Wroclaw, Fryderyka Joliot-Curie, Wroclaw, Poland; b Department of Genetics, Institute of Life Sciences, Hebrew University of Jerusalem, Jerusalem, Israel

**Keywords:** Caenorhabditis elegans, Drosophila, Emery Dreifuss Muscular Dystrophy, Lamin, laminopathies, nuclear envelope, progeria

## Abstract

Lamins are evolutionarily conserved nuclear intermediate filament proteins. They provide structural support for the nucleus and help regulate many other nuclear activities. Mutations in human lamin genes, and especially in the *LMNA* gene, cause numerous diseases, termed laminopathies, including muscle, cardiac, metabolic, neuronal and early aging diseases. Most laminopathies arise from autosomal dominant missense mutations. Many of the mutant residues are conserved in the lamin genes of the nematode *Caenorhabditis elegans* and the fruit fly *Drosophila melanogaster*. Our current understanding of the mechanisms leading to these diseases is mostly based on patients cell lines and animal models including *C. elegans* and *D. melanogaster*. The simpler lamin system and the powerful genetic tools offered by these invertebrate organisms greatly contributed to such studies. Here we provide an overview of the studies of laminopathies in *Drosophila* and *C. elegans* models.

## Laminopathies and lamins

Laminopathies are a group of diseases caused by mutations in the lamin genes [1, 2]. The mammalian genome contains 3 lamin genes. *LMNA*, which encodes lamins A and C (A-type lamins), is the most mutated lamin gene in the human genome with over 600 disease-causing mutations. Mutations in *LMNB1*, encoding lamin B1, and *LMNB2*, encoding lamin B2 (B-type lamins), also lead to diseases [3]. Laminopathies fall into five major categories. The muscle diseases include Emery Dreifuss muscular dystrophy (AD-EDMD), Limb Girdle muscular dystrophy type 1b (LGMD1b), *LMNA*-related congenital muscular dystrophy (LCMD) and dilated cardiomyopathy with conduction disease (DCM-CD). The metabolic diseases including Dunnigan-type familial partial lipodystrophy (FPLD) and several lamin mutations which are strongly linked to the metabolic syndrome [4]. The 3^rd^ category includes the peripheral neuronal disease Charcot-Marie-Tooth (AD-CMT). The 4^th^ category includes diseases affecting multiple tissues: Restrictive Dermopathy (RD), Hutchinson-Gilford progeria syndrome (HGPS), Atypical HGPS, Atypical Werner syndrome and Mandibuloacral dysplasia (MAD). The 5th group are demyelinating disorder (leukodystrophy), which is caused by overexpression of lamin B1 [3].

Lamins are nuclear intermediate filament proteins (IFs). Like all IF proteins, they are composed of a N-terminal head domain, a central alpha helical rod domain composed of 3 alpha helical coils, which are separated by linker regions, and a carboxyl tail domain [5]. In vertebrates, lamins include six heptad repeats in coil 1A, which are missing in vertebrates cytoplasmic intermediate filaments, a nuclear localization signal (NLS), an Ig fold at the tail domain and C-terminal CaaX (C = cysteine, a = aliphatic amino acid residue, X = any amino acid residue) motif. The CaaX motif undergoes posttranslational processing with an addition of a farnesyl group to the cysteine, cleavage of aaX and carboxy methylation of the farnesylated cysteine. Exceptions of lamin proteins that do not contain the CaaX motif include the lamin C isoform in vertebrates and lamin C in *Drosophila*. The vertebrate lamin A undergoes additional cleavage of 15 amino acids upstream to the farnesylated and methylated cysteine [6]. In the nucleus of human HeLa cells, lamins make a ∼14 nanometer thick meshwork under the inner nuclear membrane, which is made of 3.5 nanometer thick lamin filaments [7, 8]. A fraction of lamin A is also present in the nucleoplasm, but the assembly state of this fraction is unknown [9].

The lamin meshwork is involved in maintaining nuclear integrity and mediating mechano-signalling, as well as for regulating many other functions such as chromatin organization, cell cycle regulation, cell differentiation and signalling [10].

## Lamins in invertebrates

Every metazoan genome contains at least one lamin gene. The number of lamin genes increased in evolution. Most invertebrates, including *C. elegans*, express one B-type lamin gene, while some invertebrates express two B-type lamins [11]. The *Drosophila melanogaster* genome is so far unique in having both a B-type lamin (Dm) and an A-type lamin (LamC) genes [12–14].

The *Drosophila* lamin Dm is longer then mammalian lamin B1 (622 versus 586 residues) and is similar in length to mammalian lamin B2. It has a longer N-terminal head domain and an additional 10-residue spacer in the tail domain. Similar to mammalian B-type lamins, lamin Dm contains the cdk1/cdc2 sites flanking rod domain and its carboxyl terminus is farnesylated and methylated at the CaaX motif [15]. Lamin Dm is ubiquitously expressed throughout development. There are three known different modifications of lamin Dm: Dm_1_ and Dm_2_ have different interphase modifications and Dm_mit_ has the mitotic modifications [16]. The Dm_2_ isoform arises from Dm_1_ by phosphorylation on the N-terminal domain at or around S^25^ [2, 17]. Both Dm_1_ and Dm_2_ interact with nucleic acids *in vivo* [18]. Dm_mit_ is soluble during mitosis, presumably by phosphorylation by cdc2/cdk1 [16, 19]. *In vitro*, higher order assemblies of lamin Dm depolymerize by cdc2/cdk1, protein kinase C (PKC) or protein kinase A (PKA) [2, 20].

Reduction in the expression levels of lamin Dm was studied *in vitro* and *in vivo*. *In vitro*, antibody inhibition of lamin Dm activity in embryo cell-free assembly system inhibited nuclear envelope formation [21]. Complete genetic knockout of lamin Dm allele is lethal only at the larval/pupae stage [22]. This is probably due to the large amounts of maternally deposited lamin Dm. Partial lamin Dm gene deletions result in milder phenotypes [23]. Indeed, inhibiting the maternal pool of lamin Dm caused embryonic lethality with mitotic phenotypes. The germ line mutant clones lacking lamin Dm have abnormal dorsal-ventral polarity of the oocyte and the transcripts of the dorsal determinant Gurken fail to localize properly around the anterodorsal surface of the oocyte nucleus [24]. These results show that lamin Dm is essential for embryonic development and is required for proper mRNA positioning.

Although the *Drosophila* lamin C is probably the result of lamin Dm-like duplications [14, 25], it has many conserved features of A-type lamins including developmentally regulated expression [15, 26]. Overexpression of lamin C causes larval specific lethality [27], while lamin C knockdown results in muscle defects.

The worm *C. elegans* contains one lamin gene (*lmn-1*) encoding one lamin isoform (Ce-lamin) [28]. Ce-lamin has a head domain that is 14 amino acids longer than human lamin A. It lacks two heptad repeats in coil 2A and has a relatively short tail domain. Similar to B-type lamins, it has a CaaX box at its carboxy terminus and at lease a fraction of the protein molecules is farnesylated [29]. Similar to A-type lamins, Ce-lamin is required to maintain nuclear structure, has a small fraction present in the nucleoplasm and it interacts with proteins that in human interact with lamin A [9].

*In vitro* Ce-lamin makes stable 10 nm-wide filaments and is so far the only lamin for which the condition of filament assembly have been determined [30, 31]. The assembly of these lamin filaments starts by forming a lamin dimer. The dimers make head-to-tail polymers. Two polymers assemble laterally to form a 4 molecule wide protofilament and 3 to 4 protofilaments form the lamin filaments [32]. When injected into *Xenopus* oocytes Ce-lamin forms 4–6 nm wide filaments, which are probably in the form of protofilaments [33]. At the nuclear envelope of somatic embryonic and larval cells there are 4 nm wide filaments, which probably represent Ce-lamin protofilaments [34]. The difference in the size of filaments between HeLa cells (3.5 nm) [7] and *C. elegans* (4–6 nm) can be due to the use of the more accurate CMOS camera in HeLa or to a difference in lamin assembly between human cell line and *C. elegans* cells.

Reductions in the amount of Ce-lamin protein lead to lethality in early embryos. Other abnormalities include unequal separation of chromosomes into daughter nuclei, abnormal condensation of interphase chromatin, an increase in DNA content, and abnormal distribution of nuclear pore complexes(NPCs) [28]. Ce-lamin is also required for localizing many proteins to the nuclear periphery including the LINC complex SUN-domain protein UNC-84 and the LEM-domain proteins emerin and LEM-2 [35, 36].

## Laminopathy models in *Drosophila*

The presence of two lamin genes coding for the B-type lamin Dm and the A-type lamin C makes the fly system an attractive model to study laminopathies. An additional advantage of the fly system is that it encodes homologs of most of the vertebrate lamin interacting proteins [37, 38]. When expressed in *Drosophila* larval tissue, the human lamins A, C, B1 and/or B2 were localized mainly to the nuclear periphery. However, the human lamin C was more nucleoplasmic compared to *Drosophila* lamin C and overexpression of lamin B2 caused proliferation of both nuclear envelope membranes [39]. *Drosophila* cells that are null for lamin C showed nuclear phenotypes that resemble the defects in nuclei of patient cells expressing human lamin A mutations, including separation of the inner and outer nuclear membranes and disruptions in the nuclear envelope of imaginal discs nuclei of second instar larvae. In addition, the nuclei of larvae body wall muscle cells were irregular in shape and contained polymerized actin filaments [39]. These results show similarity in the effects of human lamin overexpression in *Drosophila*.

Missense mutations were introduced to *Drosophila* lamin C gene. Expressing high levels of the mutations N210K (N195K in *LMNA*), R401K (R386K in *LMNA*), K493W (K453W in *LMNA*), W557S (W520S in *LMNA*, which replaces evolutionarily conserved tryptophan in Ig fold) and L567P (L530P in *LMNA*) caused lethality [39]. In a second study, the laminopathy mutations: G489V (G449V in *LMNA*), N496I (N456I in *LMNA*), V528P (L489P in *LMNA*) and M553R (W514R in *LMNA*) were expressed only in the body wall muscles of fly larvae [40]. The mutant flies died when lamin C expression was driven by embryo and early larvae muscle specific promoter (Mef2) and viable when driven by an adult muscle specific promoter (MHC). The mutations V528P and M553R induced relocation of a fraction of FG-repeat nucleoporins, the NPC membrane gp210 and the SUN-domain klaroid proteins from the nucleus to the cytoplasm.

*Drosophila* strains expressing a lamin C gene lacking the first 42 residues in the head domain (headless lamin C) or the lamin C missense mutations N210K, R401K, K493W, W557S and L567P, driven by the embryo and larval muscle specific promoter, were used to test the response of muscle cells to mechanical stress in larval body wall muscle cells. Only the headless lamin C residues caused a dominant-negative effect resulting in reduced nuclear stiffness. The muscle-specific expression of lamin point mutations resulted in substantially milder disturbances in nuclear deformability, which was not statistically significant [41].

Nuclear stiffness was analyzed in the lamin C mutations G489V, N496I, V528P and M553R driven by an embryo and larval muscle-specific promoter and was compared to nuclear mechanics of wild-type lamin C and headless lamin C [41]. These laminopathic mutants demonstrated only minor changes compared to wild type lamin C. Only the headless lamin C showed a strong nuclear deformability phenotype [42].

The lamin C G489V mutation and the headless lamin C were also used to study their effects on gene expression using a GeneChip arrays. When compared to ectopic expression of wild-type *Drosophila* lamin C, the expression of the G489V mutant changed the expression of 87 genes while headless lamin C affected expression of only 28 genes. 21 of these genes, which code for proteins involved in large variety of functions, were altered in both G489V and headless lamin C. Interestingly, two of these genes: glutathione transferase and oxidoreductase are associated with oxidative/reductive stress [42]. Concomitantly, headless lamin C and G489V mutants significantly induced the level of reduced glutathione and NADPH.

Nuclear translocation of Cap-and-collar-C protein, the fly homolog of human NRF2 protein was observed in V528P and M553R mutants, as well as Keep1 proteins disappearance. Canonical NRF2/ARE pathway (Keep1-dependent) is responsible for regulation of oxidative stress and inflammatory stress response [43], which are deregulated in most laminopathies including muscle-related laminopathies and progeria. This suggests that Nrf2 pathway may contribute to the toxicity of laminopathy mutations V528P and M553R [42].

## Laminopathty models in *C. elegans*

The sequence conservation between Ce-lamin and human lamin A allows mutating residues in Ce-lamin that correspond to mutations that in human lamin A cause laminopathic diseases. This approach of introducing laminopathic mutations to Ce-lamin was used in both *in vitro* studies and *in vivo* studies [44].

### *In vitro* and ex-vivo studies

**Figure 1.**
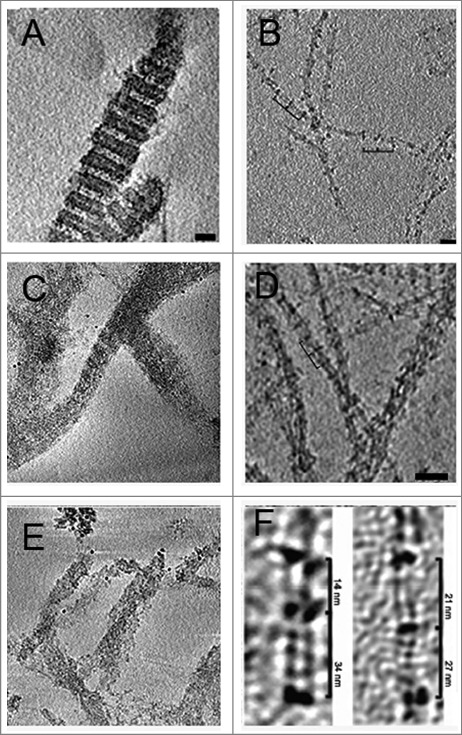
The *in vitro* structure of the *C.* *elegans* lamin filament: (A) Wildtype Ce-lamin paracrystalline arrays. (B) Wildtype Ce-lamin filaments. (C) Ce-lamin paracrystalline arrays of the progeria Q159K mutation. (D) Ce-lamin filament-like structure of the Q159K mutation. The repeat unit of the filament is different. (E) Paracrystalline arrays of Ce-lamin with the EDMD L535P mutation. While filaments apparently look like wildtype filaments (not shown) they order in the Paracrystalline arrays is lost. (F) The repeat units in Ce-lamin filament containing the EDMD DeltaK46 mutation are 34 and 14 nm compared to 21 and 27 nm in the assembled wildtype protein [45, 55]. Scale bars are 50 nm for panels A-E.

Studies of lamins’ assembly *in vitro* are performed on the bacterially expressed protein. The protein aggregates are denatured with urea and Ce-lamin is usually purified using affinity columns approaches. DNA contaminations are removed with DNAse treatment, usually using Benzonase. Filaments are assembled by dialyzing for few hours against Tris-HCl buffer at pH 9 followed by one hour dialysis against Tris-HCl buffer at pH 7. Addition of divalent ions results in paracrystalline arrays [31]. Likewise, dialysis at protein concentration of above 0.1 mg/ml results in the formation of paracrystalline arrays (unpublished observations). Using small angle X-ray scattering analysis (SAXS) on Ce-lamin hydrogels made of paracrystalline arrays of Ce-lamin showed a repeat unit of 45 nm, which probably represent the lamin rod domain (Sela, N., Beck, R., Medalia and Gruenbaum, Y. Unpublished observations). Using the above experimental approach and negative staining TEM, it was shown that 8 of 15 tested mutant lamins present wild-type-like assembly into filaments or paracrystals, whereas 7 mutants show assembly defects [41,42]. Studying mutant lamin structure at the higher resolution cryo-EM was performed on 4 mutations: Q159K (progeria; E145K in *LMNA*), T164P (EDMD, T150P in *LMNA*), L535P (EDMD, L530P in *LMNA*) and deltaK46 (EDMD, deltaK32 in *LMNA*) [45, 46] (Fig. 1). Q159K showed abnormal assembly of both filaments and paracrystalline arrays. While in wild-type filaments the repeating units of the globular tail domain are 21 nm and 27 nm (±3), the Q159K repeating units were 17 nm and 31 nm (±3) and the protofilaments assembled into thicker filaments, which tended to bundle. L535P showed filaments that resemble the wild-type filaments, but the filaments failed to produce paracrystalline arrays. Although expressing the T164P mutation cause nuclei to lobulate, both filaments and paracrystalline arrays were apparently normal. Cryo-electron tomography of lamin deltaK46 filaments *in vitro* revealed alterations in the lateral assembly of dimeric head-to-tail polymers, which causes abnormal organization of tetrameric protofilaments; the average spacing between tail domains in the deltaK46 mutant lamin alternated between 14 and 34 nm (±3), in contrast to 21 and 27 nm in wild-type lamin (Fig. 1). The recent introduction of direct electron detector Complementary metal–oxide–semiconductor (CMOS) sensor camera improve the resolution of cryo-electron tomography to less than a nanometer resolution and should allow a better resolution of yjese Ce-lamin filaments.

When expressed in *Xenopus* oocytes the Ce-lamin Q159K progeria-linked mutation formed 4–6 nm thick filaments with altered interactions between protofilaments within the lamina. The mutation also altered the flexibility of the filaments and induced some stiffness, which is apparent from the increase in persistence length (292 nm vs 167 nm in wild-type lamin). The lamin layer was found to be 30 nm thick and the Q159K Ce-lamin mutant had a high tendency to form bundled protofilaments aligned in specific directions [33], which resembles the situation in HGPS cells [47].

### Cellular studies

Transgenic animals expressing disease-linked lamin mutations were generated. The mutant lamins were expressed using the *baf-1* promoter and express 15%-30% on the normal wild-type levels and showed cellular phenotypes. Nine Ce-lamin disease-linked missense mutations, including the 7 mutations that gave *in vitro* assembly phenotypes (see above) were tested in cells and gave abnormal distribution of Ce-lamin, abnormal nuclear shape or change in lamin mobility [44]. Two mutations (R64P and R460W) did not produce viable strains and could not be tested.

*C. elegans* is a well-studied organism when it comes to determining pathways that affect life span. Studies of nuclear architecture in aging animals showed that most non-neuronal cell types undergo progressive and stochastic age-dependent alterations, such as changes of nuclear shape, loss of peripheral heterochromatin and lamin aggregation. These phenotypes are also hallmarks of cells expressing laminopathic mutations. Inhibiting farnesylation in the aging animals caused the nuclei to become round again and chromatin distribution to appear normal. However, while the age-dependent changes in nuclear morphology depended on farnesylation, they did not affect motility or lifespan, suggesting that the effects of blocking protein prenylation on nuclear morphology could be separated from their effects on motility and lifespan [48].

### Living animal studies

**Figure 2.**
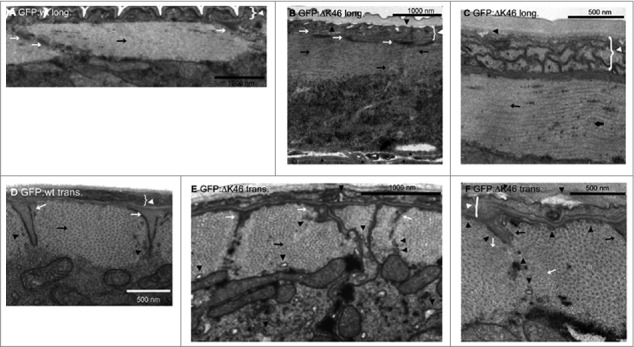
Transmission electron microscopy images reveals muscle abnormalities in animals expressing GFP:ΔK46. Longitudinal (long) (A) and transverse (trans) sections of wildtype animals or animals expressing GFP:ΔK46. Data is taken from Fig, 8 in [43].

Sixteen disease-linked lamin mutations were used to generate stable *C. elegans* strains dominantly expressing these mutations. These disease mutations represent models for the laminopathies EDMD, DCM, FPLD and HGPS. The generated strains showed dominant negative phenotypes that resemble the human laminopathies. For example, the EDMD model animals showed muscle-specific phenotypes. Expressing the deltaK36 or Y59C EDMD mutations resulted in reduced swimming motility and disordered muscle morphology (Fig. 2), while crawling motility and fertility were normal [49]. Expressing the T164P or the L535P EDMD mutations resulted also in reduced crawling motility with T164P animals showing some reduction in fertility [49, 50]. Beside body muscle phenotypes, the EDMD patients also suffer from cardiomyopathy. The *C. elegans* pharynx is a model for the heart and the pharynx muscles resemble heart muscles. So far, the pharynx activity was measured only in the L535P animals. These animals have a reduced pumping activity and overall loss of proper pharyngeal muscle structure with misorganized sarcomeres and mitochondria, which seemed to localize within sarcomeres [50]. The *C. elegans* strain expressing the progeria mutation Q159K showed progeria phenotypes including motility, fertility and early aging phenotypes [49].

### Using C. elegans to test laminopathies models

The over 14 different diseases that are caused by the *LMNA* gene and the over 600 disease-causing mutations in the lamin A protein are unprecedented among human genes. Several models were suggested which try to explain laminopathies. The gene expression model suggests that many genes are affected by mutations in lamin A, which lead to changes in chromatin organization, signalling pathways and gene expression patterns. Since many lamin binding proteins show tissue specific expression or localization [51], mutations in lamin residues that are required for the regulation of the tissue-specific genes by lamin A lead to a tissue specific phenotypes. The evolutionary conservation of Ce-lamin allowed testing the disease phenotypes in *C. elegans* animals. Over 14 disease models for laminopathies were generated [44]. Interestingly, the disease phenotypes resembled phenotypes in human laminopathy patients ([Fig. 2) 45, 46].

The effect of lamin mutation on gene positioning and gene expression was tested on the Y59C strain [52]. When expressed at low levels in otherwise wild-type worms the lamin carrying the Y59C EDMD mutation, there is an abnormal retention at the nuclear envelope of a gene array bearing a muscle-specific promoter. This was correlated with impaired activation of the array-borne myo-3 promoter and reduced expression of a number of muscle-specific genes. The tissue specificity of this effect was demonstrated by the fact that an equivalent array carrying the intestine-specific *pha-4* promoter was expressed normally and shifted inward when activated in gut cells of the Y59C worms. Therefore, lamin helps sequester heterochromatin at the nuclear envelope. Wild-type lamin permits promoter release following tissue-specific activation, while a disease-linked point mutation in lamin impairs muscle-specific reorganization of a heterochromatic array during tissue-specific promoter activation in a dominant manner, which phenocopies the autosomal dominant form of EDMD [52].

Studies of the effect of laminopathic mutations on the transcriptome were done on the L535P animals. The results showed that 960 genes were significantly downregulated and 838 genes were significantly upregulated in L535P EDMD animals. The most significant upregulated clusters involve genes of defense and stress response mechanisms. The most significant downregulated clusters involve genes of cytoskeleton organization and mitochondria function [50].

The mechanical model of laminopathies suggests that the function of lamin in maintaining nuclear integrity [28] is impaired in nuclei expressing the mutant lamin. Trials to test this model for laminopathies on isolated cells or on muscle fibers suffered from the lack of contribution of the extracellular matrix and neighboring cells and tissues [53]. *C. elegans* has the advantage that the outcome of mechanical strain application can be studied on the whole living organism [54]. Using this whole organism approach, it was shown that several laminopathic mutations affect the mechanical behaviour of some nuclei, while having no apparent effect on others. The EDMD mutation L535P disrupted the nuclear mechanical response specifically in muscle nuclei. Inhibiting lamin prenylation in the L535P animals rescued the mechanical response of the EDMD nuclei. Interestingly, inhibiting lamin prenylation also reversed the muscle phenotypes and led to normal motility of these animals [54]. The latter data implicate that *C. elegans* can be used as a whole living animal to study mechanical properties of nuclei in health and disease states and inplies that lamin-link laminopathic mutations can affect the mechanical properties of nuclei in a tissue specific manner.
